# Selected Properties of a Zr-Containing AlSi5Cu2Mg Alloy Intended for Cylinder Head Castings

**DOI:** 10.3390/ma15144798

**Published:** 2022-07-08

**Authors:** Dana Bolibruchová, Lukáš Širanec, Marek Matejka

**Affiliations:** Department of Technological Engineering, Faculty of Mechanical Engineering, University of Zilina, Univerzitná 8215/1, 010 26 Žilina, Slovakia; danka.bolibruchova@fstroj.uniza.sk (D.B.); marek.matejka@fstroj.uniza.sk (M.M.)

**Keywords:** zirconium, AlSi5Cu2Mg, cylinder head, automotive, casting

## Abstract

The aim of this paper was to analyze the impact of the addition of different amounts of zirconium (0.05; 0.10; 0.15 and 0.20 wt. % Zr in the form of the AlZr20 master alloy) on selected properties of AlSi5Cu2Mg aluminum alloy. This is a new alloy for cylinder head castings and has only been used for a relatively short time. The specificity of this alloy is its chemical composition—specifically the low permitted Ti content, which makes it impossible to refine the grain structure of this alloy using standard Al-Ti-B grain refiners. The aim of our ongoing research is to find a suitable alloying element that would positively mainly affect the mechanical and also physical properties of this alloy, which are crucial for complex automotive castings such as cylinder heads. The results of our research showed that increasing zirconium content had no effect on the increase in ultimate tensile strength, yield strength and hardness of as-cast samples. After T7 heat treatment, a more significant increase in UTS, YS and thermal conductivity occurred due to the precipitation of Cu- and Mg-rich strengthening precipitates. Zirconium-rich intermetallic phases were observed in the shape of separate thick needles or as a cluster of two crossed thinner needles. SEM observations showed that these phases crystallized near to the intermetallic phases based on Cu and Fe. Increasing the Zr content was accompanied by an increase in liquidus temperature, the density index and the area fraction of porosity values.

## 1. Introduction

In recent years, we have witnessed the automotive industry undergoing significant changes. Due to increasingly stringent regulations on emission standards, car manufacturers are forced to come up with technological solutions to reduce emissions and improve fuel efficiency. In addition to emission-improving solutions such as particulate filters, low-viscosity oils or fuels with bio additives, automakers are introducing to the market vehicles with alternative powertrains (especially hybrid and fully electric). The introduction of emission-reducing technological solutions also affects manufacturers of vehicle components (e.g., engine parts, battery trays, and electric motor housings). The above components are preferably made of aluminum alloy castings. This fact affects the research centers that have to develop new aluminum alloys that will meet the criteria for advanced automotive castings. In this way, the competitiveness and sustainability of production in the automotive sector is ensured [1,2,3].

Even though the current trend in the automotive industry is based on the promotion of electric cars, the world is far from ready for the full transition to electromobility. The high prices of electric cars, insufficient infrastructure of charging stations, lack of rare metals for battery production and the difficulties in recycling them are the factors for which fossil fuels and conventional combustion engines will not be completely displaced in the coming years [4].

**Figure 1:**
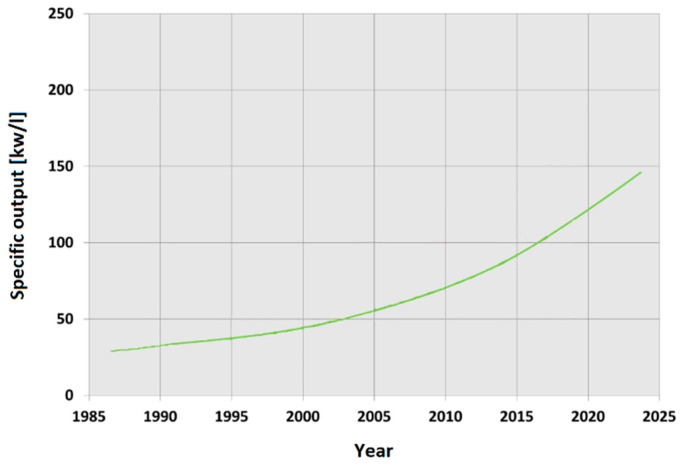
Increasing the specific output of engines [5].

Conventional internal combustion diesel/petrol engines have undergone significant changes in recent decades. The common denominator of these changes is the so-called “downsizing”. This term expresses the effort of manufacturers to provide more efficient vehicles with lower emissions by reducing engine displacement and/or number of cylinders while maintaining or even enhancing engine performance. As a result, the specific output of an engine (describing the efficiency of an engine in terms of power-to-displacement ratio) is constantly increasing (Figure 1). This leads to an increase in combustion temperatures and pressures [5,6,7,8].

Cylinder heads are among the most thermally stressed engine components. During operation, they must be able to withstand temperature changes, mechanical loads and the corrosive effects of exhaust gases. Commercially available cast aluminum alloys for cylinder heads (mostly based on the Al-Si-Cu-Mg system) can operate at temperatures up to 200 °C. Above this temperature, coarsening and dissolving of main strengthening phases (Mg_2_Si or Al_2_Cu) occurs, resulting in a decrease in the mechanical properties. Cylinder heads of modern engines are exposed to temperatures exceeding 200 °C and therefore it is necessary to focus on development of new alloys for such kind of use [9,10].

One way to prevent a decrease in mechanical properties at temperatures above 200 °C is the use of alloying elements from the group of transition metals (Zr, Ni, Mo, etc.). Of all transition metals, zirconium (Zr) has been the subject of various studies in recent years. The grain-refining effect of Zr and the thermal stability of Zr-containing intermetallic phases are attributes that predetermine its use in the development of new high-strength aluminum alloys operating at elevated temperatures above 200 °C [11,12].

### 1.1. The Grain-Refining Effect of Zr

Grain refinement of cast aluminum alloys is a basic metallurgical operation. The grain refinement mechanism is based on the introduction of inoculant particles in the form of master alloys, resulting in the nucleation initiation of α (Al) phase on the surface of these particles at small undercooling. Alloys with a finer grain structure may have improved mechanical properties, higher homogeneity and reduced possibility for hot tearing [13].

To express the effectiveness of a chemical element used as a grain refiner, a growth-restriction factor Q has been introduced. A higher value of the Q factor means that the grain refiner more effectively restricts grain growth and affects the formation of a fine-grained equiaxed structure. The growth-restriction factor can be determined as [14,15,16,17,18]:

Q = m ∙ c_0_(k_0_ − 1) [K] (1)

where m is the liquidus line gradient, c_0_ is the solute content in the alloy and k_0_ is the partition coefficient.

The Q factor value (and thus the efficiency of the grain refiner) is closely related to the segregation properties of the alloying element. Segregation properties can be described by the partition coefficient k_0_ [19], defined as:

k_0_ = c_s_/c_l_ (2)

where c_s_ and c_l_ represent the equilibrium solute contents of the solid and liquid at the growing interface.

**Figure 2:**
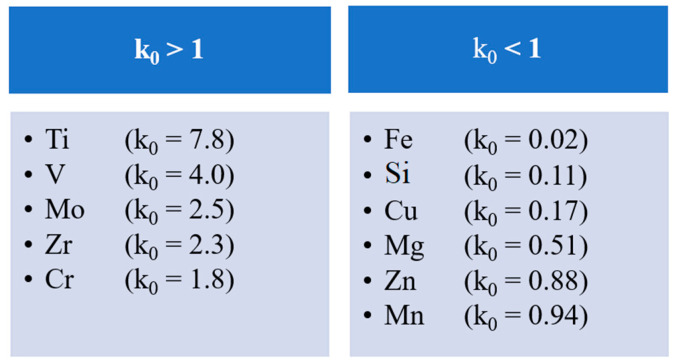
Partition coefficients (k_0_) of alloying elements [20].

If k_0_ > 1, the solute is preferentially absorbed by the growing solid rather than being segregated ahead. In areas enriched by an alloying element, heterogeneous nucleation can take place. Therefore, a potential grain-refining element in aluminum alloys should have a partition coefficient greater than 1. Such elements include (in addition to the commonly used Ti) Zr, Mo and V (Figure 2) [20,21].

### 1.2. Thermal Stability of Zr-Rich Intermetallic Phases

According to Knipling [22], aluminum alloys designed for high-temperature applications (>200 °C) should be composed of alloying element(s) that meets three basic criteria:

1. Capability of forming strengthening intermetallic phases with trialuminide type Al_3_X (X = transition metals, lanthanides or actinides);
2. Low solid solubility in Al, to maximize the equilibrium volume fraction of the dispersed phase, retard volume diffusion-controlled coarsening and prevent dissolution of precipitated phases;
3. Low diffusivity in Al, to retard volume diffusion-controlled coarsening through which the precipitates are allowed to act as an effective barrier to dislocation movement at elevated temperatures.

Zirconium as an alloying element meets all three criteria. Zr-containing aluminum alloys gained popularity due to the formation of the trialuminide Al_3_Zr phase with a cubic L1_2_ structure. The peritectic Al-Zr system is characterized by a limited concentration of Zr in the solid solution after conventional solidification [23,24,25].

Slow diffusion of the alloying element in aluminum is key to maintaining the mechanical properties or retarding their decrease at elevated temperatures. Chemical elements from the group of transition metals (Zr, Ti, Mo, V, etc.) are characterized by very low diffusion in Al; therefore, aluminum alloys alloyed with such elements are particularly suitable for the production of alloys operating at elevated temperatures. The values of diffusivity D and activation energy Q are important parameters for assessing the thermal stability of precipitates containing a transition metal. As shown in Table 1, the diffusivity values of transition metals (including Zr) at elevated temperatures are compared to, e.g., copper by 2 to 9 orders of magnitude lower, which has the effect of achieving high-temperature stability of precipitates containing such elements. High activation energy Q required to initiate diffusion expresses the intensity of precipitation coarsening at elevated temperatures—the higher the Q value, the more resistant the precipitate to coarsening and dissolution [26,27,28].

The aim of our research was to investigate the effect of varying Zr addition on selected properties of the non-standardized AlSi5Cu2Mg alloy. This alloy was developed as a result of Korean automobile concern over the production of gasoline engine cylinder head castings. As this alloy has been used in production for a relatively short time, there is minimal (if not any) available research data about this alloy. This non-standardized AlSi5Cu2Mg alloy has been designed with a very specific chemical composition—low permitted Ti content, which practically makes it impossible to grain refine the microstructure via the standard AlTi5B1 grain refiner. The aim of our research was to find suitable alloying elements that would positively mainly affect the microstructure and mechanical properties of this alloy. Zr is experiencing increasing popularity as an alloying element due to its grain-refining effect and the possibility of increasing strength characteristics of aluminum alloys. This was our motivation to address the effect of Zr on selected properties of the AlSi5Cu2Mg alloy in our research. Most studies published so far have been focused on evaluating the impact of Zr on the mechanical properties and microstructure of standardized hypoeutectic Al alloys with 7 to 10 wt. % Si. The presented research was focused on the evaluation of Zr impact in the non-standardized AlSi5Cu2Mg aluminum alloy with low Si content (5 to 6.5 wt. %). Our research deals with a more complex evaluation of the non-standardized AlSi5Cu2Mg alloy. In addition to the influence of Zr on mechanical properties and microstructure, we also dealt with the influence of Zr on thermal conductivity, melt gasification or porosity formation. Based on this, we believe our research differs from others in the greater complexity of assessing the impact of Zr on selected properties of the hypoeutectic AlSi5Cu2Mg alloy.

## 2. Materials and Methods

The hypoeutectic aluminum alloy AlSi5Cu2Mg in a pre-modified state (containing 0.01 wt. % of Sr—see Table 2) was used as an experimental material. This alloy is currently used for production of cylinder head castings. A required chemical composition of this alloy is given in Table 3. Compared to cylinder head casting alloys used so far, this alloy has several specificities:

1. Low Si content, in particular the lower limit of the Si range (Table 2). So far, cylinder head alloys, such as AlSi8Cu3, AlSi7MgCu0_._5 or AlSi10Mg(Cu), have been used and contain from 6 to 10 wt. % Si.
2. Limited Ti content (max. 0.03 wt. %). This limitation practically restricts the use of commercially available grain refiners based on Al-Ti-B. For a sufficient grain-refining effect of hypoeutectic Al-Si alloys, it is necessary to add 0.04 to 0.1 wt. % Ti.

Experimental alloys AlSi5Cu2MgZrX with varying Zr addition (0.05, 0.10, 0.15 and 0.20 wt. % Zr) were produced by the introduction of Zr in the form of the AlZr20 master alloy. Chemical composition of the experimental alloys is given in Table 3. It is important to note that the real Zr content in variants with addition of 0.15 and 0.20 wt. % Zr was lower due to impaired solubility of the AlZr20 master alloy in the melt. The amount of Zr addition in the experimental alloys was chosen based on the literature review of works dealing with Zr additions and based on our previous research on this topic at Department of Technological Engineering, University of Žilina, Slovakia.

The production of experimental alloys consisted of melting the primary alloy AlSi5Cu2Mg in an electric-resistant furnace. At 780 ± 5 °C, Zr was added in the form of the AlZr20 master alloy. The casting temperature was 745 ± 5 °C. Experimental samples were produced by gravity casting into a permanent metal mold with temperature ranging from 180 to 200 °C. Ten experimental samples were produced for each experimental alloy. Half of the samples were used for subsequent evaluation of selected properties in the as-cast state and half were heat treated by age hardening. Experimental samples were age hardened using T7 over-aging heat treatment consisting of solutioning at 500 ± 5 °C for 6.5 h, followed by quenching into hot water (80–90 °C). Artificial aging was carried out at 250 ± 5 °C for 4 h, followed by air cooling. The T7 mode is often used for heat treatment of castings with complex geometry (such as cylinder heads) to achieve a relatively stable microstructure and mechanical behavior during operation. T7-type heat treated aluminum alloys are characterized by scarification of some degree of strength to improve other characteristics. Strength characteristic are mainly “traded off” to improve dimensional stability of cylinder heads (to secure trouble-free operation at elevated temperatures) and to lower residual stresses [29,30].

**Figure 3:**
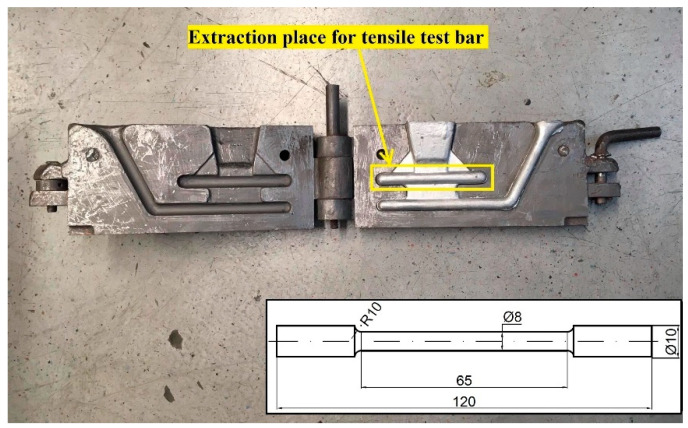
Metal mold with scheme of the tensile test bar.

Mechanical properties of the experimental alloys were evaluated based on the tensile strength test according to ISO 6892-1 standard using an Inspekt desk 50 kN testing machine. For each experimental alloy, 10 tensile test bars were produced (five for the as-cast state and five for T7 heat treatment), as shown in Figure 3.

Microstructural evaluation was performed on samples with the best combination of mechanical properties. The microstructure was observed using a NeoPhot 32 optical microscope and TESCAN LMU II scanning electron microscope with a BRUKER EDX analyzer. Samples for metallographic evaluation were prepared by coarse and fine wet grinding, two-step polishing using a 3 and 1 µm diamond paste and subsequent etching with H_2_SO_4_ etchant.

The area fraction of porosity and the length of Zr-rich intermetallic phases were evaluated using the graphic software Quick Photo Industrial 3.1. Five random places on each experimental sample were chosen to evaluate these properties.

Thermal conductivity of the experimental alloys was evaluated by the alloy manufacturer’s method. This method was based on the measurement of the electrical conductivity using a Sigma Check 2 conductometer with a touch sensor. Five measurements were performed for each experimental alloy. Obtained values of electrical conductivity (σ) were used in the empirical Formula (3) to calculate the values of thermal conductivity (λ).

Λ = 4.29 · σ − 13,321 [W·m^−1^·K^−1^] (3)

The solidification path of the experimental alloys was evaluated by thermal analysis. Equipment for the thermal analysis consisted of a K-type thermocouple located in the middle of the cylindrical shaped mold. Using a LabView 2 Hz software, measured values (temperature, time) during solidification were recorded. A cooling curve and its first derivative were generated from the obtained data.

Hardness of the experimental alloys was evaluated by Brinell method using a Brinell Innovatest Nexus 3000 hardness tester with 5 mm diameter ball with a load of 250 kp (2451.6 N) and a dwell time of 10 s. A total of 5 measurements were performed for each experimental alloy.

## 3. Results and Discussion

### 3.1. Thermal Analysis

Characteristic solidification temperatures depending on the addition of different amounts of Zr obtained from the cooling curves and their first derivatives are shown in Table 4. The above values represent the initial point in nucleation of the individual structural components.

**Figure 4:**
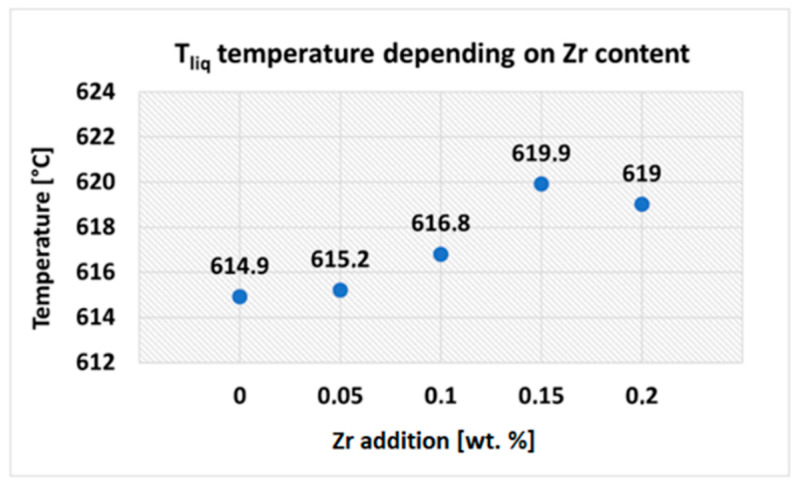
Liquidus temperature depending on Zr addition.

With increasing Zr addition, liquidus temperature (T_liq_) increased (Figure 4), reaching the highest value in the variant with 0.15 wt.% Zr addition. An increase in T_liq_ temperature (and thus widening of the solidification interval) can adversely affect the hot-tearing susceptibility of the experimental alloys.

### 3.2. Porosity Evaluation

**Figure 5:**
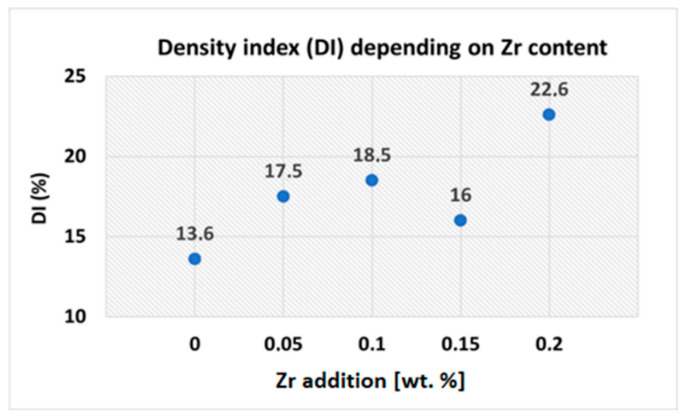
DI values of the experimental alloys.

Mechanical properties, pressure tightness and the surface quality of aluminum alloys are closely related to hydrogen solubility during melting. Hydrogen content and changes in its solubility during solidification of the alloy are closely related to the formation of porosity in castings. Intentional degassing absence of experimental alloys was carried out to study the possible impact of Zr on the density index (DI). Density index evaluation belongs to the basic procedures to evaluate the quality of the melt. Based on the DI value, it is possible to determine the degree of hydrogen gasification of the melt. DI determination was based on a density comparison of two samples, one of which solidified at atmospheric pressure and the other in vacuum (80 mbar for 4 min). It is clear from the measured values (Figure 5) that the Zr alloying process had a significant effect on DI values. An increase in DI value was already noticed in alloys with 0.05 wt. % Zr addition, where DI was 17.5%. This represents a 29% increase over the alloy without Zr addition (with DI = 13.6%). An additional increase in Zr content resulted in a further increase in DI values, with the highest value (DI = 22.6%) recorded for the alloy with addition of 0.20 wt. % Zr. One possible reason for the increase in DI values could be the metallurgical process of alloying via the AlZr20 master alloy, accompanied by a higher melting temperature and longer holding time for complete melting of the AlZr20 master alloy. These were the factors that fundamentally affected the DI values of the experimental alloys.

**Figure 6:**
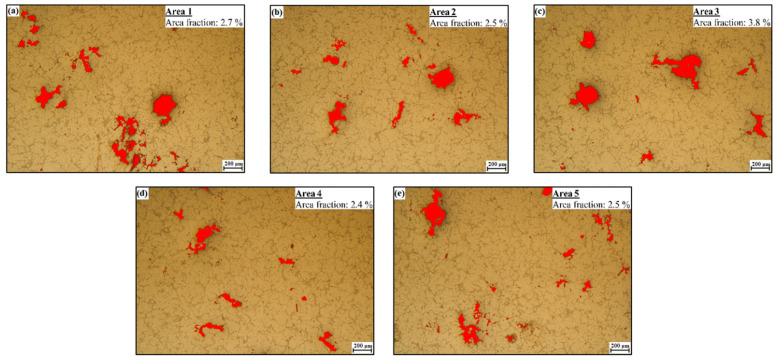
(**a**–**e**) Example of porosity evaluation by using Quick Photo Industrial 3.1 software (alloy with 0.20 wt. % Zr addition, H_2_SO_4_ etch.).

**Figure 7:**
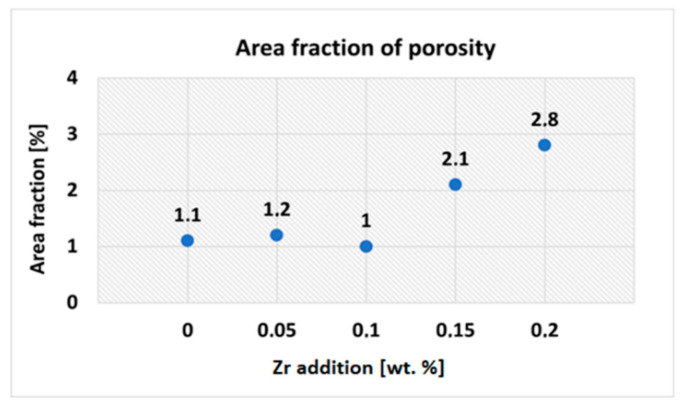
Area fraction of porosity depending on Zr addition.

The effect of Zr on porosity was also evaluated by determining the area fraction of porosity (overall shrinkage and gas pores were considered). On each experimental alloy, five different locations were selected for the measurement. The pores (red areas in Figure 6a–e) were detected using a “phase analysis” feature of the Quick Photo Industrial 3.1 software. Final values of the area fraction of porosity (Figure 7) represents the average of 5 measurements. The area fraction of porosity of the experimental alloy without Zr addition was 1.1%. Adding 0.05 and 0.10 wt. % Zr had no significant effect on porosity values. However, addition of 0.15 and 0.20 wt. % Zr resulted in an increase in the area fraction of porosity by 90 and 155%, respectively. Increased porosity of the experimental alloys after Zr addition accompanied by a reduction in the cross-sectional area of the material could have an effect on the decrease in mechanical properties. Sharp edges of the individual shrinkage pores could produce stress concentrations and could also be one of the reasons for the decrease in mechanical properties. Porosity can also be accompanied by a decrease in thermal conductivity, as the pores impede heat transfer (thermal conductivity of still air in pores is approximately four orders of magnitude lower than that of the Al matrix) [31]. Decreased thermal conductivity due to higher porosity can dramatically reduce the performance of cylinder head castings.

### 3.3. Mechanical Properties

**Figure 8:**
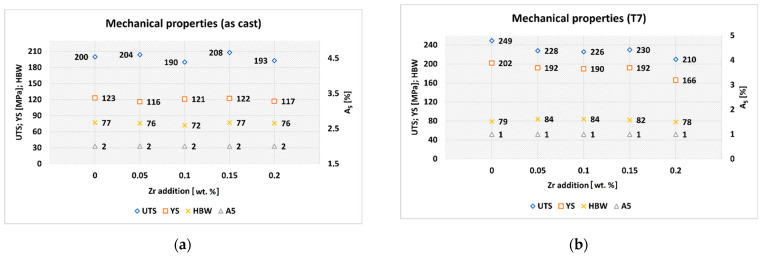
Mechanical properties of the experimental alloys AlSi5Cu2Mg: (**a**) as-cast state; (**b**) after T7 heat treatment.

Mechanical properties of the experimental alloys with varying Zr addition in the as-cast state and after T7 heat treatment are shown in Figure 8a,b. The above values represent the average of five measurements for each experimental alloy. From the obtained results it can be stated that the addition of Zr to the AlSi5Cu2Mg alloy did not significantly affect mechanical properties in the as-cast state. Based on this, it can be assumed that the grain-refining effect of Zr was not significant for the AlSi5Cu2Mg alloy. After T7 age hardening, a more significant increase in UTS and YS occurred as a result of the precipitation of Cu and Mg strengthening phases, not due to Zr addition. However, the heat-treated experimental alloys with Zr addition showed a decrease in UTS and YS compared to the heat-treated experimental alloy without Zr additive. Under the experimental conditions, it was not possible to provide sufficiently high crystallization rates for complete entry of Zr into the solid solution. This led to the formation of primary Al_3_Zr crystals, which may have caused a deterioration of the mechanical properties. Hardness values after heat treatment were slightly improved (compared to alloy without Zr addition) after the addition of 0.05 and 0.10 wt. % Zr by 6%, and after the addition of 0.15 wt. % Zr by 4%. The probable cause was crystallization of hard Al_3_Zr phases, which contributed to the overall increase in hardness. After heat treatment, ductility values decreased from 2 to 1% regardless of Zr addition.

### 3.4. Thermal Conductivity

**Figure 9:**
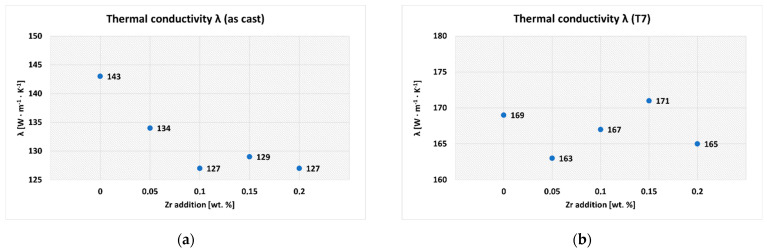
Thermal conductivity of the experimental alloys: (**a**) as-cast state; (**b**) after T7 heat treatment.

When developing new types of aluminum alloys for complex castings in the automotive industry, great emphasis is placed not only on mechanical properties but also on sufficient thermal conductivity. This is especially important in the cylinder head castings of modern turbocharged combustion engines with direct fuel injection, where the cylinder heads can be exposed to temperatures exceeding 200 °C during operation. In order to achieve optimal engine temperature during operation, it is necessary to address the impact of individual alloying elements on thermal conductivity of newly developed alloys. The impact of varying Zr addition in the AlSi5Cu2Mg alloy on thermal conductivity in as-cast and heat-treated state is shown in Figure 9a,b. These values represent the average of five measurements for each experimental alloy.

When evaluating the thermal conductivity of experimental alloys in the as-cast state, it was found that the presence of Zr resulted in a 6 to 11% (depending on Zr addition) decrease in thermal conductivity in all variants. From the thermal conductivity point of view, Zr acts as an “impurity”, because the addition of any other element to the alloy has an effect on the decrease in thermal conductivity. Thermal energy in alloys is transferred due to the vibration of atoms and free (delocalized) electron movement. Excited atoms in one section works to excite and vibrate adjacent atoms. This motion (or kinetic energy) allows heat to move through the alloy. Any alloying element will affect the free electron path and the vibrational state of the surrounding atoms, resulting in a change in thermal conductivity [32,33]. This was also a probable cause of the reduction in thermal conductivity of experimental alloys alloyed with Zr.

**Figure 10:**
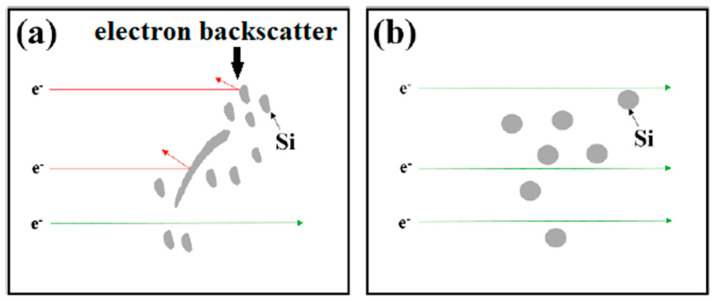
Electron movement in aluminum alloy: (**a**) as-cast; (**b**) heat treated.

After T7 heat treatment, thermal conductivity of alloy without Zr addition increased by 18%. Depending on the changing Zr addition, thermal conductivity increased by 22 to 33% in Zr-alloyed experimental alloys. However, it should be noted that the addition of Zr in any amount did not have a significant effect on the change in thermal conductivity after heat treatment compared to the alloy without Zr. Thus, it can be stated that the addition of Zr in the AlSi5Cu2Mg alloy did not have a negative effect on the significant reduction in thermal conductivity. This is why it is possible to use such Zr-containing alloy in the development of a new alloy intended for the automotive industry (especially the production of cylinder heads). Thermal conductivity of the experimental alloys in the over-aged T7 state was probably improved due to the formation of fine, uniformly distributed precipitates, which depleted the matrix of solute [17]. Spheroidization and clustering of eutectic Si particles also played important role in thermal conductivity, whereas more favorable conditions for electron movement (as thermal energy carriers) were created (see Figure 10) [34].

### 3.5. Microstructure

**Figure 11:**
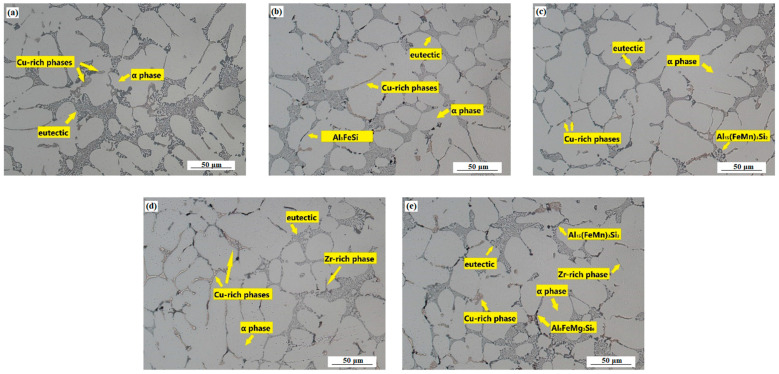
Microstructure of the experimental alloys AlSi5Cu2MgZrX (as-cast, H_2_SO_4_ etch.): addition of (**a**) 0 wt. % Zr; (**b**) 0.05 wt. % Zr; (**c**) 0.10 wt. % Zr; (**d**) 0.15 wt. % Zr; (**e**) 0.20 wt. % Zr.

Depending on Zr addition, the microstructure of experimental alloys in the as-cast state (Figure 11a–e) consisted of α phase, modified eutectic Si and intermetallic phases based on Cu, Mg, Fe and Zr.

**Figure 12:**
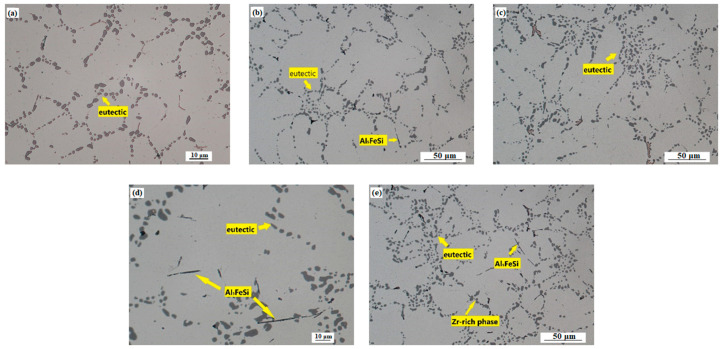
Microstructure of experimental alloys AlSi5Cu2MgZrX (T7, H_2_SO_4_ etch.): addition of (**a**) 0 wt. % Zr; (**b**) 0.05 wt. % Zr; (**c**) 0.10 wt. % Zr; (**d**) 0.15 wt. % Zr; (**e**) 0.20 wt. % Zr.

After T7 heat treatment, dissolution of Cu- and Mg-rich intermetallic phases occurred during solution heat treatment, further creating small, more uniformly dispersed precipitate particles. This was the reason for the significant increase in mechanical properties (see Figure 8a,b. Due to the T7 heat treatment, spheroidization and clustering of eutectic Si particles occurred (Figure 12a–e). Formation of strengthening precipitates together with spheroidization of eutectic Si particles played an important role in increasing thermal conductivity of the experimental alloys after heat treatment, creating more favorable conditions for thermal energy carriers.

**Figure 13:**
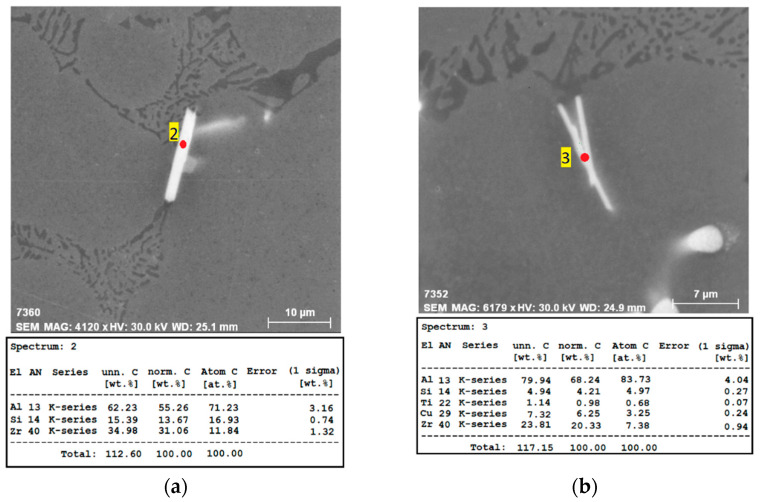
Morphology of Zr phases in the AlSi5Cu2Mg alloy with the corresponding EDX spectrum (as-cast, H_2_SO_4_ etch.): (**a**) 0.20 wt. % Zr addition; (**b**) 0.15 wt. % Zr addition.

Zr-rich intermetallic phases were observed in shape of separated thicker needles (Figure 13a) or as a cluster of two thinner crossed needles (Figure 13b). These phases were observed only in alloys with addition of 0.15 and 0.20 wt. % Zr. There was no evidence of these phases in alloys with addition of 0.05 and 0.10 wt. % Zr. The probable cause was the low real content of Zr in these alloys, which did not exceed the maximum solubility of Zr in the solid solution and therefore no Zr-rich intermetallic phases were formed during solidification. The average length of Zr phases was 19 µm (SD = 1.9) for the alloy with addition of 0.15 wt. % Zr and 14 µm (SD = 1.1) for the alloy with addition of 0.20 wt. % Zr.

**Figure 14:**
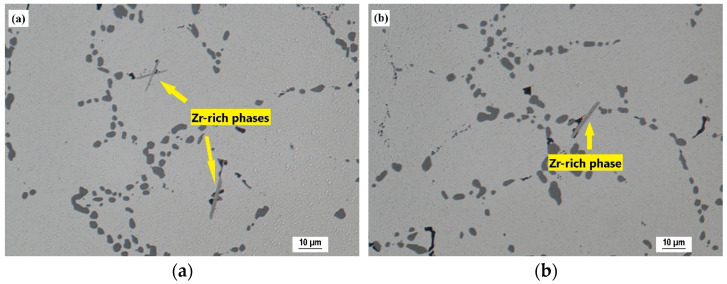
Presence of Zr-rich intermetallic phases after T7 heat treatment (H_2_SO_4_ etch.): (**a**) 0.20 wt. % Zr addition; (**b**) 0.15 wt. % Zr addition.

**Figure 15:**
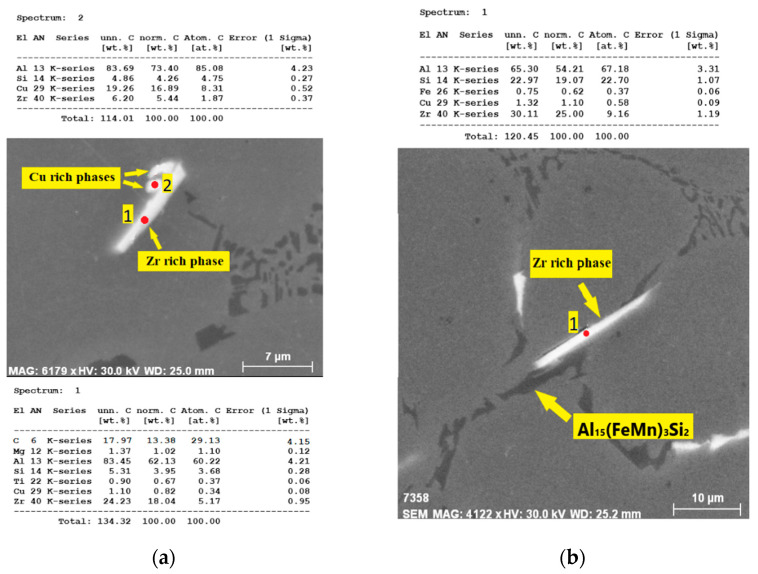
Interaction of: (**a**) Zr and Cu phases (0.15 wt. % Zr addition, as-cast, H_2_SO_4_ etch.); (**b**) Zr and Fe phases (0.20 wt. % Zr addition, as-cast, H_2_SO_4_ etch.).

T7 heat treatment did not affect the Zr-rich intermetallic phases. Such needle-shaped phases (Figure 14a,b) were observed without any changes in their morphology or length compared to the as-cast state. This indicates high thermal stability of these phases, which were not affected even at elevated temperatures during solution heat treatment. Microstructural observations using SEM with EDX analysis were performed in order to identify possible interaction of Zr-rich phases with other intermetallic phases. It was found that Zr-rich phases with acicular morphology were observed near Cu-rich phases (Figure 15a) and adjacent to complex Fe-Mn-Si-rich phases, such as Al_15_(FeMn)_3_Si_2_ with morphology of segmented skeletal structures (Figure 15b).

## 4. Conclusions

The aim of this work was to investigate the effect of graded Zr addition to the non-standardized AlSi5Cu2Mg alloy in order to create a new alloy that could be potentially used in the production of complex castings for the automotive industry (such as cylinder heads). The most important findings are:

- The addition of Zr resulted in an increase in the liquidus temperature and thus in the overall widening of the solidification interval.
- The significant increase in DI values and the area fraction of porosity was noticed with increasing Zr content.
- The addition of Zr to the AlSi5Cu2Mg alloy via the AlZr20 master alloy did not have significant effect on mechanical properties in the as-cast state. After T7 age hardening, a more significant increase in UTS and YS occurred as a result of the precipitation of Cu and Mg strengthening phases, not due to Zr addition. However, the heat-treated experimental alloys with Zr addition showed a decrease in UTS and YS compared to the heat-treated experimental alloy without Zr additive. One possible cause could be the formation of primary Al_3_Zr crystals with tetragonal D0_23_ crystal structure, which may have caused a decrease in mechanical properties. Further research on crystal structure of the Al_3_Zr phase in the experimental alloys will be needed. However, it can be concluded that the addition of Zr caused deterioration in mechanical properties of the experimental alloys.
- Zr addition had an effect on reducing the thermal conductivity of experimental alloys in the as-cast state. Thermal conductivity of the heat-treated experimental alloys with Zr addition was almost unchanged compared to the heat-treated experimental alloy without Zr addition. This suggests that the addition of Zr did not have significant adverse effect on the thermal conductivity of the experimental alloys.
- Zr-rich intermetallic phases were present in experimental alloys with the addition of 0.15 and 0.20 wt. % Zr with morphology of separated thicker needles or as a cluster of two thinner crossed needles. There was no presence of these phases in experimental alloys with the addition of 0.05 and 0.10 wt. % Zr. Such Zr-rich phases were present without change in morphology after T7 heat treatment. This was indicating their high thermal stability at elevated temperatures.
- SEM observations with EDX phase analysis revealed the crystallization of Cu and Fe phases adjacent to Zr-rich intermetallic phases, indicating that Zr phases created suitable nucleation sites for other intermetallic phases.

From the results obtained during this experiment, it can be concluded that addition of Zr in the AlSi5Cu2Mg alloy showed a deterioration of the mechanical properties. The decrease in mechanical properties was probably related to the insufficient cooling rate during solidification, which led to the formation of primary Al_3_Zr crystals. The production of aluminum alloys with Zr addition requires accelerated cooling to provide complete entry of Zr into the solid solution, resulting in the formation of a supersaturated solid solution, which subsequently provides an increase in strength properties.

## Figures and Tables

**Table 1:** Diffusivity and activation energy values of selected transition metals [22].

Element	Q [kJ∙mol^−1^]	D at 400 °C [m^2^∙s^−1^]
Sc	173	1.98 × 10^−17^
Ti	260	7.39 × 10^−22^
Zr	242	1.20 × 10^−20^
Mo	250	5.52 × 10^−23^
V	303	4.85 × 10^−24^
Cr	282	1.29 × 10^−21^
Cu	136	1.54 × 10^−15^

**Table 2:** Chemical composition of the experimental alloys AlSi5Cu2MgZrX.

Chemical Composition [wt. %]									
Zr Addition [wt. %]	Si	Cu	Mg	Fe	Mn	Ti	Sr	Zr	Al
0	5.47	1.91	0.29	0.18	0.02	0.013	0.01	0.0009	Bal.
0.05	5.67	1.91	0.29	0.19	0.02	0.013	0.01	0.05	Bal.
0.10	5.65	1.92	0.29	0.19	0.02	0.014	0.01	0.10	Bal.
0.15	5.55	1.91	0.29	0.19	0.02	0.014	0.01	0.12	Bal.
0.20	5.43	1.90	0.29	0.18	0.02	0.014	0.01	0.19	Bal.

**Table 3:** Required chemical composition of the AlSi5Cu2Mg alloy.

Alloy	Chemical Composition [wt. %]							
**AlSi5Cu2Mg**	Si	Fe	Cu	Mn	Mg	Sr	Ti	Al
	5.0–6.5	max. 0.20	1.6–2.5	max. 0.03	0.15–0.35	0.008–0.012	max. 0.03	Bal.

**Table 4:** Characteristic solidification temperatures of the AlSi5Cu2MgZrX alloys.

Zr Addition [wt. %]	T_liq_	T_Al-Si_	T_Mg2Si_	T_Al2Cu_	T_sol_
0	614.9	565.5	555.6	503.4	517.3
0.05	615.2	564.5	554.9	504.6	517.2
0.10	616.8	565.9	554.8	502.1	516.9
0.15	619.9	565.3	554.4	500.1	517.6
0.20	619.0	564.9	554.3	500.0	517.4

## Data Availability

Data available on request. The data presented in this study are available on request from the corresponding author.
